# Transcriptome sequencing reveals the roles of transcription factors in modulating genotype by nitrogen interaction in maize

**DOI:** 10.1007/s00299-015-1822-9

**Published:** 2015-06-27

**Authors:** Qiuyue Chen, Zhipeng Liu, Baobao Wang, Xufeng Wang, Jinsheng Lai, Feng Tian

**Affiliations:** National Maize Improvement Center of China, China Agricultural University, Beijing, 100193 China

**Keywords:** G × N interaction, Nitrogen response, RNA-seq, Transcription factor, *Zea mays*

## Abstract

****Key message**:**

**Global transcriptome analysis in maize revealed differential nitrogen response between genotypes and implicate a crucial role of transcription factors in driving genotype by nitrogen interactions at gene expression level.**

**Abstract:**

Developing nitrogen-efficient cultivars are essential for sustainable and productive agriculture. Nitrogen use efficiency of plants is highly dependent on the interaction of environmental and genetic variation and results in adaptive phenotypes. This study used transcriptome sequencing to perform a comprehensive genotype by nitrogen (G × N) interaction analysis for two elite Chinese maize inbreds grown at normal and low nitrogen levels in field conditions. We demonstrated that the two maize inbreds showed contrasting agronomic and transcriptomic responses to changes in nitrogen availability. A total of 96 genes with a significant G × N interaction were detected. After characterizing the expression patterns of G × N interaction genes, we found that the G × N interaction genes tended to show condition-specific differential expression. The functional annotations of G × N interaction genes revealed that many different kinds of genes were involved in G × N interactions, but a significant enrichment for transcription factors was detected, particularly the AP2/EREBP and WRKY family, suggesting that transcription factors might play important roles in driving G × N interaction at gene expression level for nitrogen response in maize. Taken together, these results not only provide novel insights into the mechanism of nitrogen response in maize and set important basis for further characterization but also have important implications for other genotype by stress interaction.

**Electronic supplementary material:**

The online version of this article (doi:10.1007/s00299-015-1822-9) contains supplementary material, which is available to authorized users.

## Introduction

Nitrogen (N) is a major nutritional factor limiting plant growth. Over the past few decades, heavy use of nitrogen fertilizers have played a key role in increasing crop yields, however, only 30–40 % of the applied N was actually utilized by crops (Kant et al. 2011; Xu et al. 2012). More than 60 % of the soil N is lost through surface runoff, denitrification, volatilization and microbial consumption (Kant et al. 2011; Xu et al. 2012). This loss is costly and detrimental to the environment (Kant et al. 2011; Xu et al. 2012). Thus, improving the nitrogen use efficiency (NUE) of crops is of key importance for sustainable and productive agriculture.

NUE, defined as the total biomass or grain yield produced per unit of applied fertilizer N, is a complex quantitative trait that depends on a number of internal and external factors, including soil nitrogen availability, nitrogen uptake, assimilation, transportation and remobilization (Kant et al. 2011; Masclaux-Daubresse et al. 2010; Xu et al. 2012). Considerable efforts have been made to investigate the genetic, biochemical and enzymatic mechanisms for how plants use nitrogen throughout their life span (Kant et al. 2011; Simons et al. 2014; Stitt et al. 2002; Xu et al. 2012). A number of biosynthetic enzymes, transcription factors and kinases have been found to be involved in nitrogen uptake, assimilation and remobilization (Kant et al. 2011; Masclaux-Daubresse et al. 2010). The nitrate transporters *NRT1.1*, *NRT1.2*, *NRT2.1*, and *NRT2.2* are responsible for nitrate uptake from the environment (Ho et al. 2009; Miller et al. 2007). Glutamine synthetase (GS)/glutamate synthase (GOGAT) cycle is predominantly responsible for assimilating ammonium into amino acids (Lam et al. 1996; Xu et al. 2012). Notably, overexpression of *GS1*-*3* in maize can lead to an increase of 30 % in kernel number (Martin et al. 2006). A large number of quantitative trait loci (QTLs) for physiological and agronomic traits have been identified in maize using quantitative genetic approaches to associate metabolic functions and agronomic traits to DNA markers (Agrama et al. 1999; Hirel et al. 2007; Kant et al. 2011). Previous studies have found QTL for grain yield and yield components overlapping the location of genes for N metabolism (Gallais and Hirel 2004; Hirel et al. 2001).

Next generation sequencing technology provides an unprecedented opportunity to characterize transcriptome-wide responses to environmental changes. An increasing number of transcriptome sequencing studies on maize development under different N conditions have been performed to identify N-responsive genes and regulatory control of the expression patterns (Amiour et al. 2012; Humbert et al. 2013; Simons et al. 2014). Results from these studies have shown that the transcriptional response to nitrogen availability is highly complex, contingent on a variety of developmental, metabolic, and regulatory factors (Amiour et al. 2012; Humbert et al. 2013; Simons et al. 2014). The recent transcriptome-wide studies further showed that different maize genotypes responded differently to nitrogen availability (Bi et al. 2014; Zamboni et al. 2014). These results suggested that there is wide variation of genotype by nitrogen (G × N) interaction at gene expression level. However, a further understanding of how maize genotypes interact with different N levels at transcriptional level is lacking. Studies that are specifically designed to identify genes with significant G × N interaction and characterize their regulatory features are needed in maize. Dissecting genotype by environment interactions at the transcriptional level has started to become an important approach for dissecting complex traits and understanding traits evolution (Cubillos et al. 2014; Degenkolbe et al. 2009; Des Marais et al. 2012, 2013, 2015; Geng et al. 2013; Grishkevich and Yanai 2013; Idaghdour and Awadalla 2012; Lasky et al. 2014; Laudencia-Chingcuanco et al. 2011; Lowry et al. 2013; Richards et al. 2012; Snoek et al. 2013).

In this study, using transcriptome sequencing, we performed a comprehensive genotype by nitrogen (G × N) analysis for two maize inbreds Zheng58 and Chang7-2, the parents of Zhengdan958, a maize hybrid with the largest planting area in China. The previous investigation of nitrogen use efficiency for 27 representative Chinese inbreds has shown that both Zheng58 and Chang7-2 are nitrogen-efficient inbreds at both normal and low nitrogen levels compared to other inbreds (Cui et al. 2013). However, in the response sensitivity, Chang7-2 showed a relatively greater differential response between nitrogen conditions than Zheng58 (Cui et al. 2013). The objectives of this study were to examine the transcriptomic responses to nitrogen changes in Zheng58 and Chang7-2, and further identify genes with significant G × N effects and characterize their expression patterns and functional features. We showed that Zheng58 and Chang7-2 showed a contrasting agronomic and transcriptomic responses to the nitrogen treatments. Transcription factors were significantly enriched among genes with significant G × N interactions, which implicates that transcription factors might play a crucial role in modulating the G × N interactions at transcriptional level.

## Materials and methods

### Plant materials

Zheng58 and Chang7-2 were grown in 2011 at the Shangzhuang experimental station of China Agricultural University in Beijing under normal nitrogen (NN) and low nitrogen (LN) conditions. The NN treatment indicates the application of the general agronomic fertility treatment (450 kg/ha urea). While for the LN treatment, no nitrogen fertilizer was applied. The LN experiments were conducted in locations where nitrogen fertilizer was not applied during the preceding 2 years. A total of four genotype-condition combinations, namely NN_Zheng58, NN_Chang7-2, LN_Zheng58 and LN_Chang7-2, were tested. In NN and LN field, Zheng58 and Chang7-2 were planted in seven replications. In each replication, Zheng58 and Chang7-2 were adjacently planted in single-row plot, with 10 plants per row, 25 cm between plants within each row and 50 cm between rows.

Previous studies have shown that flowering time is a critical period bridging N uptake and N assimilation during vegetative growth to post-flowering N absorption and remobilization (Hirel et al. 2007). Leaves above the primary ear act as one of the main N source for grain-filling (Crawford et al. 1982). For each genotype-treatment combination, when 80 % of plants in the plot flowered, the leaf above the primary ear was sampled for RNA sequencing. Of the seven field plot replications, for each genotype–nitrogen combination, two plot replications were randomly selected and sampled to make biological replications for RNA sequencing. The leaves above the primary ear from four randomly selected plants in the same plot replications were pooled together to make a biological replication. Samples were collected and stored at −80 °C in preparation for RNA extraction.

At the same time, a total of 15 agronomic traits were measured for each genotype-treatment combination, including plant height, ear height, leaf length, leaf width, tassel length, tassel branch number, days to anthesis, days to silking, cob length, ear diameter, kernel row number, cob diameter, cob weight, total kernel weight and hundred kernel weight. For each genotype–nitrogen combination, all seven filed plot replicates were measured for each trait, with each biological replicate having five randomly selected plants scored. The phenotypic mean of the five plants was used as the phenotype of each replication for the phenotypic difference comparison. *T* test was performed to test the significance of phenotypic difference.

### RNA sequencing and data analysis

Total RNA was isolated and purified using RNAprep pure Plant Kits (TIANGEN BIOTECH). Approximately 15 μg of total RNA was used for library construction following a standard procedure. Libraries were sequenced with a read length of 100 bp (paired-end) and an insertion size of 300 bp on an Illumina HiSeq 2000 at Berry Genomics, Beijing. Read quality was evaluated using FastQC software (Andrews 2010). 3′ reads with quality less than 20 were first trimmed by NGS QC Toolkit (v2.3) (Patel and Jain 2012). Only reads with a read length greater than 50 bp were kept for downstream analysis. The high-quality reads were then aligned to the B73 reference sequence (AGPv2) (Schnable et al. 2009) using Tophat2/Bowtie1 (Kim et al. 2013). Five mismatches, a minimum intron size of 5 bp and a maximum intron size of 60,000 bp were used for alignment. For each sample, the number of reads covering the gene model (filtered gene set 5b) was calculated using htseq-count with the intersection-strict option (Anders et al. 2014).

### Identifying genes with significant G × N interactions

To identify genes with a differential nitrogen response between genotypes (namely G × N interaction) in expression level, the R-bioconductor package “edgeR” (v3.4.0) (Robinson et al. 2010) was used to conduct the differential expression analysis. Compared to other differential expression analysis software packages, edgeR employs a robust negative binominal distribution to account for biological variation and dispersion from all genes (Rapaport et al. 2013). Only genes with at least one read count in each sample were kept for further analysis. edgeR first calculates scaling factors for the library sizes that enter into the statistical model for normalizations computed by *calcNormFactors* function. Then, edgeR uses the *model.matrix* function to construct the design matrix and estimate the BCVs and dispersions of the negative binomial model by *estimateGLMCommonDisp* and *estimateGLMTagwiseDisp* function. At last, edgeR uses *glmFit* function to fit the model and uses *glmLRT* to test the significance of differential expression for different contrasts. The G × N interaction contrast that can be simply expressed as “(LN_Zheng58-NN_Zheng58)-(LN_Chang7-2-NN_Chang7-2)” was tested for each expressed gene using edgeR. The contrasts for the expression difference between genotypes under each treatment (NN_Zheng58-NN_Chang7-2; LN_Zheng58-LN_Chang7-2) and the expression difference between treatments for each genotype (LN_Zheng58-NN_Zheng58; LN_Chang7-2-NN_Chang7-2) were also conducted for characterizing the expression patterns of G × N interaction genes. The *P* value of differential expression was converted to false discovery rate (FDR) using Benjamini and Hochberg’s algorithm (Benjamini and Hochberg 1995). Expression was considered to be significantly different at a threshold of FDR <0.1. Principle component analysis (PCA) was performed by *prcomp* and plotted by *plot3d* function in R.

### Functional annotation of G × N interaction genes

G × N interaction genes were evaluated for common functions using GO term enrichment test in AgriGO (Du et al. 2010) (http://bioinfo.cau.edu.cn/agriGO/). GO categories were considered significantly enriched with a FDR <0.05 and at least five genes in the category.

The potential functions of the identified G × N interaction genes were first analyzed using the annotation information from maizeGDB database (Lawrence et al. 2004) (http://www.maizegdb.org/) and then using the TAIR database (Swarbreck et al. 2008) (http://arabidopsis.org/) by protein BLAST. MapMan (Thimm et al. 2004) was also used to examine metabolic pathways and other biological processes.

Maize transcription factors were downloaded from the transcription factor database GrassTFDB (Yilmaz et al. 2009) (http://www.grassius.org/grasstfdb.html). Fisher’s exact test was used to test if G × N interaction genes showed significant overrepresentation of transcription factors compared to the global expressed gene sets.

### Quantitative real-time PCR analysis

First-strand cDNA synthesis was synthesized using TransScript^®^ One-Step gDNA Removal and cDNA Synthesis SuperMix (TransGene Biotech) and then stored at −20 °C for subsequent analysis. qRT-PCR was performed with the Toolkit for SYBR^®^ Green I with ROX Reference Dye II (Takara Biotechnology). Each PCR reaction contained 10 μl mixture, consisting of 1 μl cDNA, 5 μl of SYBR^®^ Green Premix Ex Taq II, 0.2 μl of ROX Reference Dye II, and 1 μl of the forward and reverse primers. All qRT-PCRs were performed in three technical replicates in 7500 Real-Time PCR System and performed in two steps: pre-denaturation for 30 s at 95 °C and 40 cycles of denaturation for 15 s at 95 °C, and annealing/extension for 34 s at 60 °C. After the PCR, a melting curve was generated by gradually increasing the temperature to 95 °C to test the amplification specificity. Outliers were manually discarded and the housekeeping gene *Actin* was used as internal standard to calculate the relative expression level for all target genes using comparative $\text{C}_{\text {T}}\left(2^{-\Delta \Delta \text{C}_{\text{T}}}\right)$ method (Schmittgen and Livak 2008).

## Results and discussion

### Transcriptional and phenotypic response for nitrogen changes

About 10.3 million clean paired-end reads were generated for each of the eight RNA-seq samples and aligned to B73 reference genome (AGPv2) (Schnable et al. 2009). On average, 81.4 % of reads were mapped to the reference genome and 82.4 % of them could be uniquely mapped (Table 1). Only uniquely mapped reads were used in subsequent analyses. For each gene model, read counts were calculated using htseq-count (Anders et al. 2014).

**Table 1:** Summary for RNA-Seq reads mapping

Sample	# Trimmed reads	Mapped reads^a^	% Mapped reads^b^	Unique reads^c^	% Unique reads^d^
NN_Zheng58 rep1	11,711,873	9,654,313	82.4	8,015,675	83.0
NN_Zheng58 rep2	12,024,837	9,679,882	80.5	8,249,117	85.2
LN_Zheng58 rep1	10,026,796	8,203,604	81.8	7,027,655	85.7
LN_Zheng58 rep2	11,961,259	7,747,455	64.8	5,801,233	74.9
NN_Chang7-2 rep1	7,524,720	6,731,286	89.5	5,394,971	80.1
NN_Chang7-2 rep2	9,888,498	7,761,912	78.5	5,929,391	76.4
LN_Chang7-2 rep1	11,991,698	10,241,108	85.4	8,840,129	86.3
LN_Chang7-2 rep2	7,137,723	6,314,603	88.5	5,546,868	87.8
Average	10,283,426	8,291,770	81.4	6,850,630	82.4

^a^Number of reads that were mapped to the B73 genome

^b^% of reads that were mapped to the B73 genome out of the total number of trimmed reads

^c^Number of uniquely mapped reads out of the total number of mapped reads

^d^% of uniquely mapped reads out of the total number of mapped reads

**Fig. 1:**
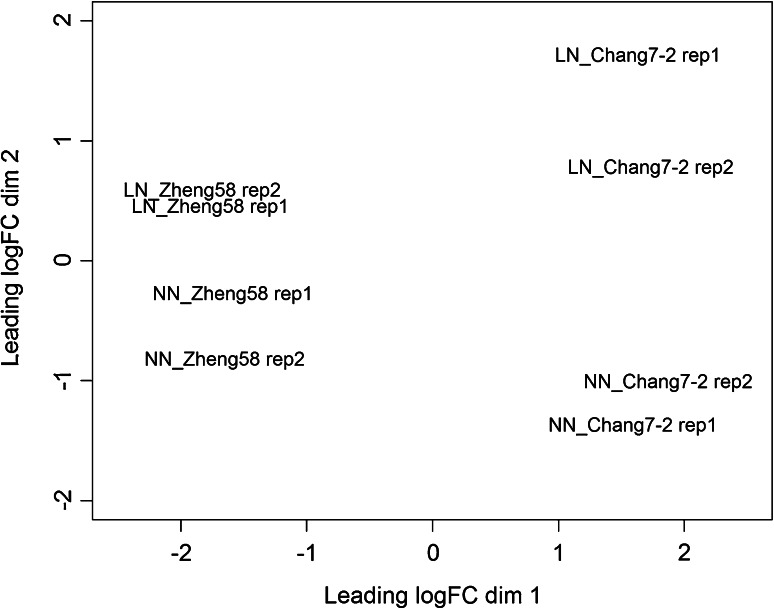
A multidimensional scaling (MDS) analysis for experimental samples. Samples separated by genotype (Zheng58 and Chang7-2) in the first dimension and by the nitrogen condition (NN and LN) in the second dimension

A total of 20,685 genes with at least one read for each sample were retained for downstream analyses. Comparisons of biological replicates showed that their expression values across all expressed genes were highly correlated (average *R*^2^ = 0.97). A multidimensional scaling (MDS) analysis was conducted using expression levels normalized by edgeR to evaluate the repeatability of biological replicates (Robinson et al. 2010). As shown in Fig. 1, biological replicates for the same genotype-treatment combination clustered together, indicating that the transcriptional variation between replicates was low relative to the variation due to genotype and treatment. The first MDS dimension separated the samples by genotype (Zheng58 and Chang7-2) and then by the nitrogen condition (NN and LN) in the second dimension. The principle component analysis (PCA) further showed that the experiment was well controlled (Fig. S1).

**Fig. 2:**
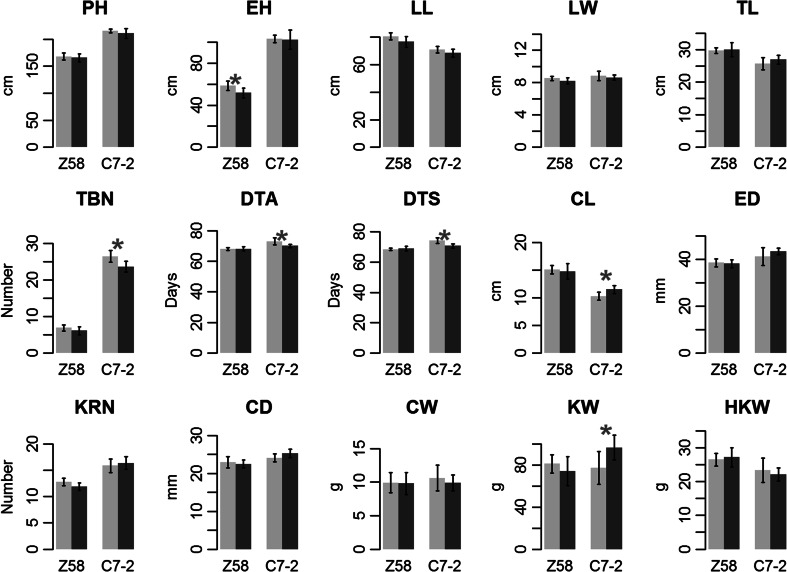
Contrasting phenotypic differences in response to nitrogen conditions between Zheng58 and Chang7-2. The phenotypic values represent mean ± SD (*n* = 7). NN and LN are indicated by *light grey* and *dark grey*, respectively. *Red asterisks* indicate a significant phenotypic difference between NN and LN (Student’s *t* test; *P < 0.05). Z58: Zheng58; C7-2: Chang7-2. *PH* plant height, *EH* ear height, *LL* leaf length, *LW* leaf width, *TL* tassel length, *TBN*, tassel branch number, *DTA* days to anthesis, *DTS* days to silking, *CL* cob length, *ED* ear diameter, *KRN*, kernel row number, *CD* cob diameter, *CW* cob weight, *KW* total kernel weight, *HKW* hundred kernel weight

Interestingly, the MDS analysis suggested that Zheng58 and Chang7-2 showed different sensitivities to the nitrogen treatments. The distance between Chang7-2 samples at NN and LN was much larger than that of Zheng58, suggesting Chang7-2 exhibited a greater transcriptional response to differences in nitrogen availability. This is consistent with the organismic-level phenotypic response of the inbreds. The plots of Zheng58 and Chang7-2 from which the RNA-seq samples were collected were evaluated for 15 agronomic traits (Fig. 2). Five traits showed significant treatment effects for Chang7-2, while only one trait differed for Zheng58.

Gene expression is an important molecular phenotype that links genetic variant and organismic phenotype. The consistent environmental response pattern between gene expression level and organismic-level agronomic traits suggest that the transcriptional level response might play key roles in determining the organismic-level phenotypic response in response to environmental changes. Therefore, the global transcriptional response of genotypes can be used as a robust predicator of their phenotypic changes in response to environmental cues. Despite the consistent global environmental response pattern between gene expression level and organismic-level agronomic traits, it is hard to construct specific links between G × N genes and the associated agronomic traits with current limited information. Further genetic dissection in segregating population is needed to establish the causal link.

It is worth noting that our study is with limitation because only one tissue and one developmental stage from a single field season were examined. The further investigations across multiple tissues and developmental stages from multiple field seasons will provide a full picture of how transcriptional variation interacts with environment to produce organismic-level nitrogen response.

### Genes with significant G × N interactions and their expression patterns

**Fig. 3:**
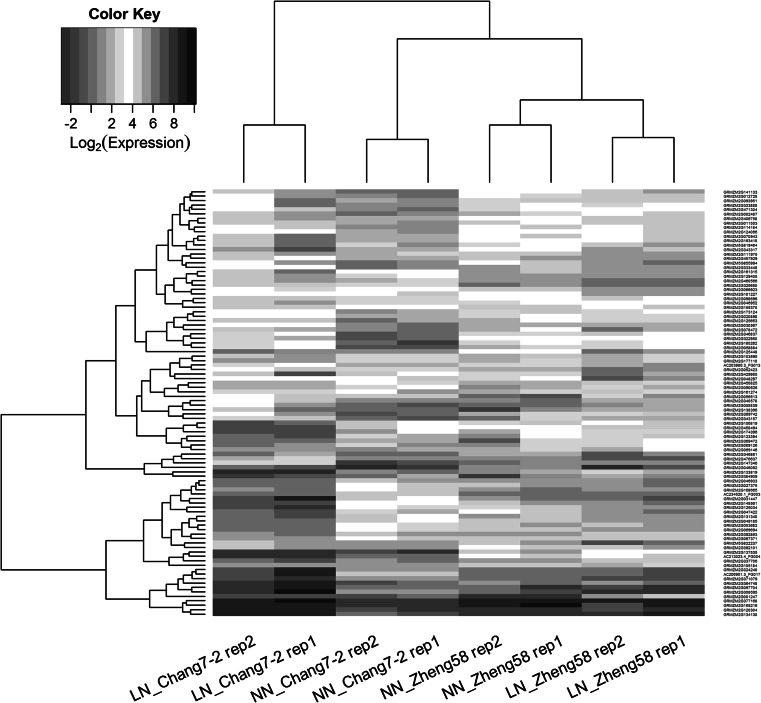
The expression heat map of 96 G × N interaction genes with dendrogram added. Rows and columns correspond to log_2_(expression) of genes and samples, respectively. *Red* and *blue* indicate lower and higher expression levels, respectively

**Fig. 4:**
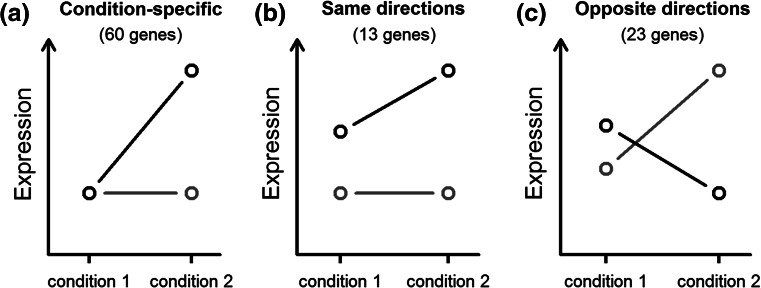
Expression patterns of G × N interaction genes. Two genotypes are indicated by *black* and *red lines*. **a** The expression difference between the genotypes is condition-specific, namely significant expression difference between the genotypes is only detected in one condition. **b** The expression differences between the genotypes are detected in both conditions and the effect direction is in the same direction in the two conditions. **c** The expression differences between the genotypes are detected in both conditions but the effect direction is in the opposite direction in the two conditions

A total of 96 genes were identified with a significant G × N interaction at FDR <0.1 (Table S1) using edgeR (Robinson et al. 2010) and their overall expression patterns are shown in Fig. 3. G × N interactions can be attributable to changes in magnitude or direction of effect. The expression profiles of the 96 G × N interaction genes across genotypes and treatments could be classified into three main patterns (Fig. 4). In pattern 1, which includes 60 genes (62.5 %), the expression difference between genotypes was only observed under either the NN or the LN treatment (Fig. 4a). For pattern 2, the expression difference between genotypes is in the same direction at both N levels (Fig. 4b), whereas for pattern 3, the expression difference between genotypes is opposite in the two conditions (Fig. 4c). A total of 13 (13.5 %) and 23 (24.0 %) genes fall into pattern 2 and 3, respectively. Of the 96 G × N interaction genes, 80 (83.3 %) genes exhibited a greater expression difference between N levels in Chang7-2 compared to Zheng58. This observation is consistent with the above transcriptome-wide analysis that showed a greater sensitivity of the Chang7-2 transcriptome to nitrogen availability.

It is worth noting that, compared to 20,685 investigated genes, the number of genes showing G × N interaction is small. This is mainly because (1) both Zheng58 and Chang7-2 are nitrogen-efficient inbreds (Cui et al. 2013). Therefore, the genetic difference in nitrogen response between Zheng58 and Chang7-2 might not be substantial, and (2) only two biological replicates were included for each genotype-condition combination. This limitation might significantly affect the statistical power to identify more G × N genes. Further investigations on inbreds with substantial nitrogen response difference and including sufficient biological replications will help identify more G × N interaction genes.

To validate the RNA-seq results, a total of six genes were selected for qRT-PCR analysis using the same samples as RNA-seq. The primer sequences used in qRT-PCR were listed in Table S2. The comparative CT method relies upon the assumption that the efficiency of the PCR is close to 1, and the target gene and internal control gene have similar PCR efficiencies (Schmittgen and Livak 2008). The very similar shapes of PCR amplification plots (Fig. S2) suggested that the investigated genes and the internal control gene *Actin* have similar PCR efficiency. As shown in Figure S3, G × N interactions detected by RNA-seq demonstrated correspondence with results obtained by qRT-PCR (*R*^2^ = 0.6, *P* = 0.067).

### Functional features of G × N interaction genes

The functional features of 96 G × N interaction genes were annotated based on the annotation information from maizeGDB, MapMan and AgriGO (Table S3–S5). The analysis showed that the 96 G × N interaction genes belong to a wide range of functional categories, including biosynthetic enzymes, transcription factors, genes involved in hormone metabolism and stress-responsive genes, which is consistent with the diverse functions previously found to underlie genotype by environment interaction (Des Marais et al. 2013). Of the 96 G × N genes, 24 genes encode biosynthetic enzymes that are involved in a number of primary and secondary metabolic processes, such as amino acid, lipid, photosynthesis, hormone metabolism and protein degradation. Similarly, Bi et al. (2014) also detected numerous genes involved in various metabolic pathways that contribute to the differential nitrogen response among three genotypes. Hormone genes that are involved in the metabolism of abscisic acid, auxin, and cytokinins have been frequently identified as important N-responsive genes in previous studies (Kiba et al. 2011). These results suggested that the changes in nitrogen limitation have triggered complex transcriptional response at diverse biological processes. Despite the wide range of functional classes of G × N genes, of 96 G × N interaction genes, 21 genes are transcription factors, which is a significant enrichment compared to the background gene set (*P* = 3.20e−05; Table 2). These transcription factors belong to 15 different types of transcription factor families. Of them, five genes belong to AP2/EREBP family and three genes belong to WRKY family and these two families showed significant enrichments (Table 2).

**Table 2:** The number of TFs across the maize genome and their enrichments in G × N interaction genes

Type	# in G × N genes list^a^	# expressed in GrassTFDB^b^	*P* value
Total number of TFs	21	1477	3.20e−05^**^
*AP2/EREBP*	5	96	1.31e−04^**^
*WRKY*	3	85	8.49e−03^**^
Total number of genes	96	20,685	

*TF* transcription factors

** Significant at *P* < 0.01

^a^Number of transcription factors showing significant G × N interactions

^b^Number of expressed transcription factors (transcription factors were annotated by GrassTFDB database)

**Fig. 5:**
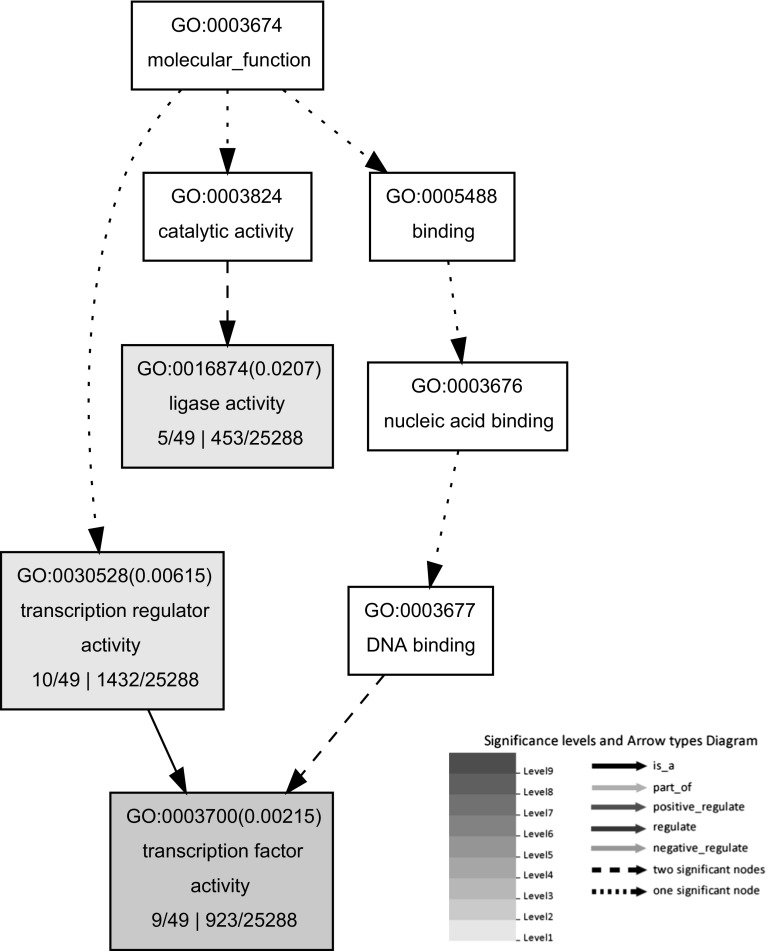
The most significantly enriched GO terms for 96 G × N interaction genes. *Boxes* in the graph represent GO terms labeled by their GO ID, term definition and statistical information

The Gene Ontology analysis has been widely used as an important approach to characterize the biological process, cellular component and molecular function of differentially expressed genes. The 96 G × N genes were found to be involved in 42, 15 and 11 GO terms in biological process, cellular component and molecular function, respectively. These wide GO categories of G × N genes are consistent with the above annotation of G × N genes. The enrichment analyses of GO terms revealed some common functional features shared by the G × N interaction genes. A total of 22 GO terms were found to be significantly enriched for the 96 G × N interaction genes at FDR <0.05, such as “regulation of metabolic process”, “regulation of nitrogen compound metabolic process”, “regulation of transcription”, “transcription factor activity” and “transcription regulator activity”. Of these significant GO terms, the most significant term is “transcription factor activity” (*P* = 6.50e−05, Fig. 5; Table S4).

Taken together, these results suggest that a number of genes with different functions have been involved in modulating the G × N interaction at transcriptional level, indicating the complexity of the molecular mechanism of G × N interaction. However, the overrepresented transcription factors in G × N genes suggested that transcription factors might play an important role in mediating the transcriptional changes in response to changes in nitrogen availability. The important roles of transcription factors in regulating plant responses to various stresses have been well demonstrated in numerous studies (Chen and Zhu 2004). Transcription factors generally contain multiple cis-regulatory elements as well as multiple DNA-binding domains. This feature provides sufficient flexibility for environmental context-dependent regulations, thus enabling transcription factors to be more easily disposed to form G × E interactions.

### Important G × N interaction candidate genes

*AP2/EREBP* and *WRKY* family proteins have been shown to play important roles in plant growth and development throughout the plant life cycle, especially in responses to different biotic and abiotic stresses (Eulgem et al. 2000; Kizis et al. 2001). *GRMZM2G177110* is a homolog of the LBD (LATERAL ORGAN BOUNDARIES DOMAIN) transcription factor *LBD37* in Arabidopsis*. LBD37* and other two close homologs, *LBD38* and *LBD39,* have been shown to be negative regulators of N availability signals, as well as of anthocyanin biosynthesis in Arabidopsis (Rubin et al. 2009). The *LBD* genes also repress many other known N-responsive genes, including key genes required for NO_3_^−^ uptake and assimilation (Rubin et al. 2009). Further characterizing *GRMZM2G177110* will provide cues for understanding the roles of the LBD gene family in maize nitrogen response. Four G × N interaction genes encode ubiquitin E3 ligases. The ubiquitin-26S proteasome pathway has been shown to play an important role in N remobilization during leaf senescence for grain-filling (Liu et al. 2008). E3 ligases ‘ubiquitinate’ target genes and thus determine substrate specificity (Zhang and Xie 2007). Nitrogen limitation adaptation (NLA), a RING-type ubiquitin E3 ligase, is a well characterized gene which has been shown to control the adaptability of *Arabidopsis* to nitrogen limitation (Peng et al. 2007). *GRMZM2G078472* encodes an asparagine synthetase (AsnS). Asparagine has been shown to play a central role in nitrogen transport and storage in plants due to its high nitrogen/carbon ratio and stability (Gaufichon et al. 2010). Three G × N interaction genes encode ABA-responsive protein. *GRMZM2G471304* encodes an auxin responsive protein. *GRMZM2G392101* is a cytokinin response regulator. These hormone genes are important for many plant growth, and developmental processes and response to environmental factors. It has also been shown that, amongst phytohormones, abscisic acid, auxin, and cytokinins have been closely linked to nitrogen signaling (Kiba et al. 2011). Four genes, including *GRMZM2G429955*, *GRMZM2G155216*, *GRMZM2G134130* and *GRMZM2G046092*, are involved in photosynthesis. The alterations in the expression of genes encoding proteins involved in photosynthesis have been shown as an important differential response when N is limiting (Amiour et al. 2012). These genes are promising candidates for further investigations of the molecular basis of nitrogen response. Identification of the specific mutations that drive G × N interactions will provide a deeper understanding of basis of phenotypic changes for nitrogen response in maize.

## Conclusions

We performed differential expression analysis of two elite maize inbreds under two field nitrogen conditions via transcriptome sequencing and identified a set of 96 genes that showed a significant genotype by nitrogen treatment interaction. Analysis of the expression patterns of these genes indicated that genes with G × N interactions were more likely to show condition-specific differential expression. Transcription factors, particularly the *AP2/EREBP* family and *WRKY* family, showed significant enrichments in G × N interaction genes, suggesting the importance of these transcription factor families in the differential nitrogen response between genotypes. Taken together, these results provide novel insights into the mechanism of nitrogen response in maize and provide a set of nitrogen responsive genes for further characterization.

### Availability of supporting data

The data set supporting the results of this article is available in the Sequence Read Archive (http://www.ncbi.nlm.nih.gov/sra/) with the accession number ‘SRP052559’. All data sets supporting the results of this article are included within the article.

#### **Author contribution statement**

QC carried out data analysis. ZL and BW carried out the field planting, management and phenotyping. ZL, QC and XW performed tissue sampling and RNA preparation. JL provided the materials. FT and JL conceived of and supervised the study. QC wrote the manuscript draft, FT edited and revised the manuscript. All authors read and approved the final manuscript.

## Electronic supplementary material

Supplementary material 1 (DOCX 639 kb)
