# Supplementary material for: Transcriptome sequencing reveals the roles of transcription factors in modulating genotype by nitrogen interaction in maize
Source: Plant Cell Rep. 2015 Jun 27;34(10):1761–71. doi: 10.1007/s00299-015-1822-9 (PMC4569664; doi:10.1007/s00299-015-1822-9)
Supplement: Supplementary file 1 — Supplementary material 1 (DOCX 639 kb) [file 299_2015_1822_MOESM1_ESM.docx]

Plant Cell Reports

Supplementary information for:

**Transcriptome sequencing reveals the roles of transcription factors in modulating genotype by nitrogen interaction in maize**

Qiuyue Chen^†^ · Zhipeng Liu^†^ · Baobao Wang · Xufeng Wang · Jinsheng Lai · Feng Tian

^†^ These authors contributed equally to this work.

National Maize Improvement Center of China, China Agricultural University, Beijing 100193, China

Correspondence and requests for materials should be addressed to Feng Tian (ft55@cau.edu.cn)

**Fig. S1** Principle Component Analysis (PCA) for experimental samples


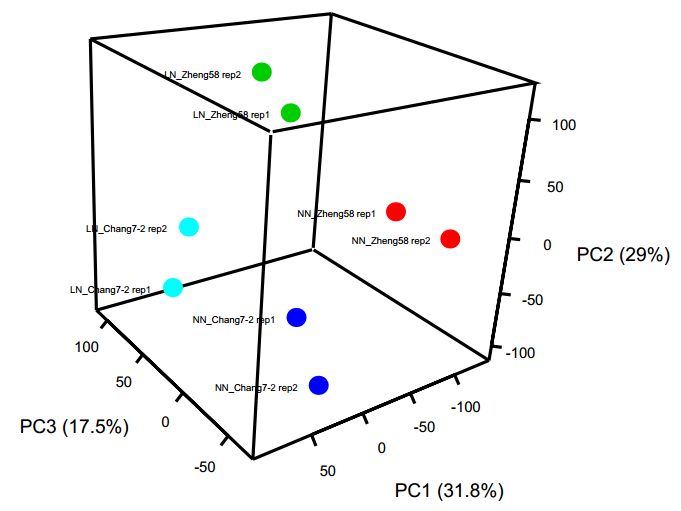


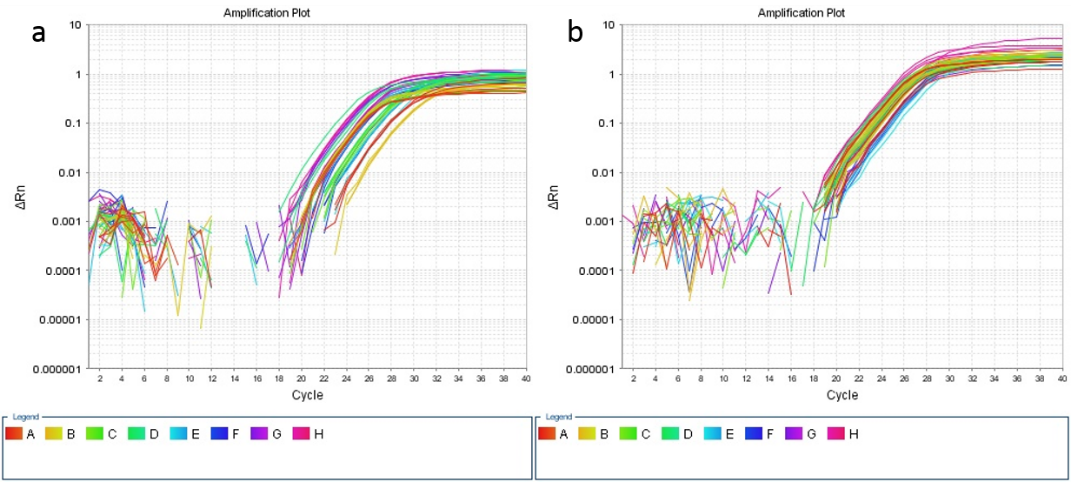
**Fig. S2 L**ogarithmic PCR amplification plots of qRT-PCR assay

**Fig. S3** The correlation of values measuring G×N interactions between RNA-seq and qRT-PCR


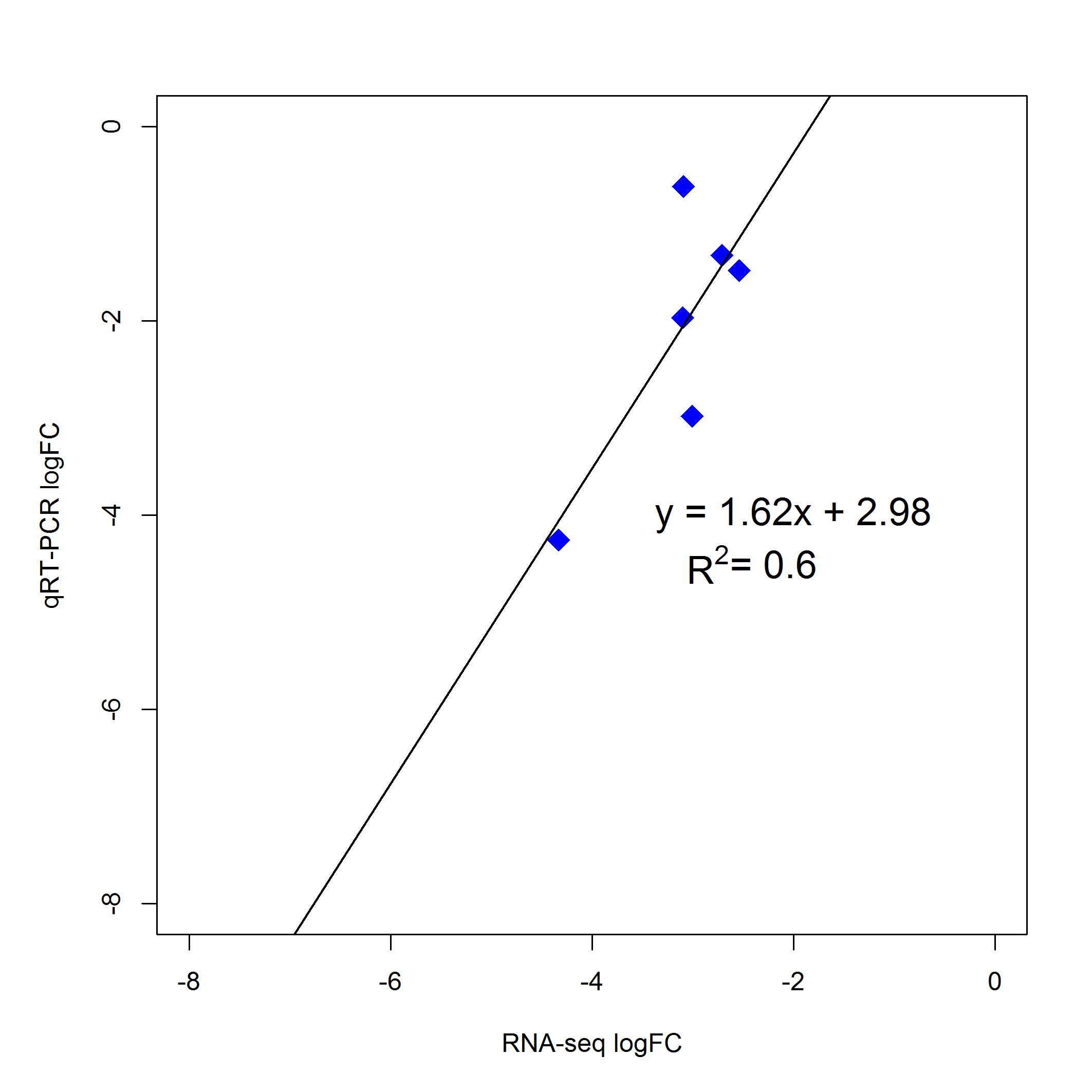


**Table S1** The list of 96 G×N interaction genes at FDR<0.1

| Gene id | logFC | logCPM | LR | PValue | FDR |
| --- | --- | --- | --- | --- | --- |
| GRMZM2G001247 | -3.42 | 6.97 | 59.13 | 1.47E-14 | 3.05E-10 |
| GRMZM2G141133 | -4.00 | 4.25 | 37.63 | 8.56E-10 | 8.85E-06 |
| GRMZM2G180262 | -4.00 | 2.97 | 27.97 | 1.23E-07 | 6.02E-04 |
| GRMZM2G471304 | -3.16 | 3.41 | 27.90 | 1.28E-07 | 6.02E-04 |
| GRMZM2G030567 | -3.22 | 2.62 | 27.65 | 1.46E-07 | 6.02E-04 |
| GRMZM2G406758 | -2.47 | 4.11 | 26.80 | 2.26E-07 | 7.80E-04 |
| GRMZM2G173124 | -2.99 | 3.29 | 25.11 | 5.40E-07 | 1.60E-03 |
| GRMZM2G069694 | -2.06 | 5.19 | 24.82 | 6.30E-07 | 1.63E-03 |
| GRMZM2G105184 | -1.93 | 4.89 | 23.59 | 1.19E-06 | 2.73E-03 |
| GRMZM2G177110 | -2.48 | 3.84 | 23.14 | 1.51E-06 | 3.12E-03 |
| GRMZM2G148561 | -2.42 | 6.07 | 22.29 | 2.35E-06 | 4.42E-03 |
| GRMZM2G333448 | -2.72 | 4.43 | 21.72 | 3.16E-06 | 5.06E-03 |
| GRMZM2G069146 | -3.08 | 4.03 | 21.70 | 3.18E-06 | 5.06E-03 |
| GRMZM2G003682 | -2.58 | 5.36 | 21.02 | 4.54E-06 | 6.27E-03 |
| AC203986.3_FG013 | -2.65 | 2.74 | 21.02 | 4.55E-06 | 6.27E-03 |
| GRMZM2G323888 | -3.82 | 3.93 | 20.17 | 7.08E-06 | 9.15E-03 |
| GRMZM2G129405 | -1.94 | 5.01 | 19.92 | 8.07E-06 | 9.82E-03 |
| GRMZM2G111975 | -1.90 | 4.63 | 19.38 | 1.07E-05 | 1.23E-02 |
| AC234520.1_FG003 | -1.78 | 5.96 | 19.05 | 1.27E-05 | 1.38E-02 |
| GRMZM5G822237 | 2.26 | 5.70 | 18.76 | 1.49E-05 | 1.54E-02 |
| GRMZM2G163418 | -2.70 | 4.23 | 17.98 | 2.23E-05 | 2.15E-02 |
| GRMZM2G056513 | 2.79 | 3.69 | 17.91 | 2.32E-05 | 2.15E-02 |
| GRMZM2G125653 | -3.44 | 3.24 | 17.76 | 2.51E-05 | 2.15E-02 |
| GRMZM2G174396 | -2.71 | 5.79 | 17.72 | 2.56E-05 | 2.15E-02 |
| GRMZM2G343317 | -2.95 | 5.04 | 17.69 | 2.60E-05 | 2.15E-02 |
| GRMZM2G070943 | -2.87 | 4.35 | 17.51 | 2.86E-05 | 2.17E-02 |
| GRMZM2G460566 | -2.04 | 5.25 | 17.51 | 2.86E-05 | 2.17E-02 |
| GRMZM2G134130 | -1.97 | 8.22 | 17.46 | 2.93E-05 | 2.17E-02 |
| GRMZM2G048297 | -2.67 | 3.37 | 17.01 | 3.71E-05 | 2.65E-02 |
| GRMZM2G392101 | 1.61 | 4.97 | 16.79 | 4.18E-05 | 2.88E-02 |
| AC206951.3_FG017 | -2.56 | 6.98 | 16.69 | 4.39E-05 | 2.88E-02 |
| GRMZM2G046952 | -2.31 | 4.05 | 16.66 | 4.48E-05 | 2.88E-02 |
| GRMZM2G429955 | -3.85 | 4.74 | 16.61 | 4.59E-05 | 2.88E-02 |
| GRMZM2G047422 | -2.71 | 5.27 | 16.50 | 4.87E-05 | 2.93E-02 |
| GRMZM2G082487 | -2.53 | 3.83 | 16.47 | 4.95E-05 | 2.93E-02 |
| GRMZM2G337706 | -2.07 | 5.99 | 16.31 | 5.37E-05 | 3.09E-02 |
| GRMZM2G181227 | 1.72 | 4.30 | 16.25 | 5.54E-05 | 3.09E-02 |
| GRMZM2G161315 | -1.87 | 5.09 | 16.19 | 5.74E-05 | 3.09E-02 |
| GRMZM2G078472 | -4.26 | 2.58 | 16.16 | 5.82E-05 | 3.09E-02 |
| GRMZM5G819464 | -2.92 | 4.74 | 16.09 | 6.03E-05 | 3.12E-02 |
| GRMZM2G377168 | -1.94 | 9.11 | 15.99 | 6.38E-05 | 3.22E-02 |
| GRMZM2G011553 | -1.84 | 3.97 | 15.81 | 7.01E-05 | 3.35E-02 |
| GRMZM2G106819 | -2.30 | 5.29 | 15.79 | 7.07E-05 | 3.35E-02 |
| GRMZM2G450825 | 3.22 | 2.92 | 15.74 | 7.28E-05 | 3.35E-02 |
| GRMZM2G098696 | -1.59 | 3.91 | 15.73 | 7.32E-05 | 3.35E-02 |
| GRMZM2G012728 | -2.91 | 3.87 | 15.69 | 7.45E-05 | 3.35E-02 |
| GRMZM2G392863 | -1.90 | 5.32 | 15.62 | 7.75E-05 | 3.41E-02 |
| GRMZM2G103595 | -2.98 | 3.82 | 15.53 | 8.12E-05 | 3.43E-02 |
| GRMZM2G058884 | -2.90 | 2.68 | 15.51 | 8.19E-05 | 3.43E-02 |
| GRMZM2G147346 | -3.38 | 1.51 | 15.49 | 8.29E-05 | 3.43E-02 |
| GRMZM2G052423 | -2.28 | 2.87 | 15.42 | 8.59E-05 | 3.45E-02 |
| GRMZM2G476637 | -3.21 | 1.41 | 15.39 | 8.74E-05 | 3.45E-02 |
| GRMZM2G340578 | -2.65 | 2.82 | 15.33 | 9.05E-05 | 3.45E-02 |
| GRMZM2G458494 | -1.89 | 5.24 | 15.32 | 9.07E-05 | 3.45E-02 |
| GRMZM2G123394 | -3.68 | 5.16 | 15.30 | 9.18E-05 | 3.45E-02 |
| GRMZM2G138396 | -3.07 | 3.73 | 15.06 | 1.04E-04 | 3.85E-02 |
| GRMZM2G131340 | -1.70 | 5.62 | 15.01 | 1.07E-04 | 3.87E-02 |
| GRMZM2G066923 | 1.74 | 4.52 | 14.95 | 1.10E-04 | 3.94E-02 |
| GRMZM2G369472 | -2.99 | 4.72 | 14.74 | 1.23E-04 | 4.33E-02 |
| GRMZM2G343157 | -3.93 | 2.74 | 14.65 | 1.29E-04 | 4.45E-02 |
| GRMZM2G031447 | -2.35 | 6.65 | 14.51 | 1.39E-04 | 4.73E-02 |
| GRMZM2G354909 | 4.14 | 1.18 | 14.47 | 1.42E-04 | 4.74E-02 |
| GRMZM2G125034 | -2.91 | 6.13 | 14.40 | 1.48E-04 | 4.86E-02 |
| GRMZM2G322950 | -2.53 | 3.16 | 14.33 | 1.54E-04 | 4.97E-02 |
| GRMZM2G008839 | -3.26 | 3.01 | 14.27 | 1.58E-04 | 5.03E-02 |
| GRMZM2G069126 | -2.51 | 4.21 | 14.19 | 1.65E-04 | 5.17E-02 |
| GRMZM2G114184 | -2.15 | 3.70 | 14.01 | 1.82E-04 | 5.61E-02 |
| GRMZM2G161274 | 3.17 | 3.72 | 13.87 | 1.96E-04 | 5.96E-02 |
| GRMZM2G346837 | -2.32 | 3.17 | 13.66 | 2.20E-04 | 6.48E-02 |
| GRMZM2G027375 | -1.57 | 5.58 | 13.63 | 2.23E-04 | 6.48E-02 |
| GRMZM2G364748 | -1.93 | 7.06 | 13.59 | 2.27E-04 | 6.48E-02 |
| GRMZM2G137535 | -2.22 | 6.85 | 13.59 | 2.27E-04 | 6.48E-02 |
| GRMZM2G020898 | -2.03 | 3.05 | 13.58 | 2.29E-04 | 6.48E-02 |
| GRMZM2G049185 | -1.42 | 5.28 | 13.55 | 2.32E-04 | 6.48E-02 |
| GRMZM2G325650 | -1.42 | 5.21 | 13.45 | 2.45E-04 | 6.76E-02 |
| GRMZM2G168665 | -1.82 | 5.45 | 13.34 | 2.60E-04 | 7.07E-02 |
| GRMZM2G155370 | -1.73 | 4.29 | 13.24 | 2.75E-04 | 7.38E-02 |
| GRMZM5G855994 | -2.81 | 4.65 | 13.11 | 2.94E-04 | 7.71E-02 |
| GRMZM2G097704 | -2.06 | 7.80 | 13.11 | 2.94E-04 | 7.71E-02 |
| GRMZM2G125448 | 2.36 | 4.10 | 13.04 | 3.05E-04 | 7.87E-02 |
| GRMZM2G369742 | -3.04 | 3.37 | 12.99 | 3.13E-04 | 7.99E-02 |
| AC212023.4_FG004 | -2.09 | 6.56 | 12.88 | 3.32E-04 | 8.30E-02 |
| GRMZM2G324248 | -2.18 | 6.83 | 12.86 | 3.35E-04 | 8.30E-02 |
| GRMZM2G093951 | -2.72 | 4.36 | 12.85 | 3.37E-04 | 8.30E-02 |
| GRMZM2G457929 | -2.09 | 4.53 | 12.81 | 3.46E-04 | 8.30E-02 |
| GRMZM2G346861 | -3.04 | 1.50 | 12.80 | 3.46E-04 | 8.30E-02 |
| GRMZM2G046933 | -1.36 | 5.64 | 12.77 | 3.51E-04 | 8.30E-02 |
| GRMZM2G155216 | -2.46 | 8.65 | 12.76 | 3.55E-04 | 8.30E-02 |
| GRMZM2G046092 | -4.11 | 0.38 | 12.75 | 3.57E-04 | 8.30E-02 |
| GRMZM2G067371 | -1.34 | 4.83 | 12.67 | 3.72E-04 | 8.56E-02 |
| GRMZM2G308595 | -2.33 | 7.83 | 12.62 | 3.81E-04 | 8.67E-02 |
| GRMZM2G133819 | 3.59 | 1.44 | 12.53 | 4.01E-04 | 9.01E-02 |
| GRMZM2G371079 | -1.37 | 6.66 | 12.51 | 4.05E-04 | 9.01E-02 |
| GRMZM2G090626 | 2.00 | 2.43 | 12.37 | 4.36E-04 | 9.44E-02 |
| GRMZM2G120304 | -1.57 | 8.13 | 12.37 | 4.37E-04 | 9.44E-02 |
| GRMZM2G124066 | -1.81 | 3.39 | 12.36 | 4.38E-04 | 9.44E-02 |

**Table S2** The primer sequence used in qRT-PCR

| Gene | Description | Forward primer (5'→3') | Reverse primer (5'→3') |
| --- | --- | --- | --- |
| GRMZM2G078472 | AsnS (asparagine synthetase) | TGCGCTTCGAGATCTTCCC | TGAGCCTCTTGATAACCGCC |
| GRMZM2G069126 | ereb23 (AP2-EREBP-transcription factor 23) | GAGACGACGGCTACACCAAT | GTGCTCTGTTTTGGGGAGTC |
| GRMZM2G069146 | ereb115 (AP2-EREBP-transcription factor 115) | AGATGTGCCCAACCAAGAAG | GTGCTCCGGTAAGGTCGAG |
| GRMZM2G138396 | ereb211 (AP2-EREBP-transcription factor 211) | GGACACCGAGACACTGACG | CTGTAGACCTTCCCACAGCTC |
| GRMZM2G369472 | ereb172 (AP2-EREBP-transcription factor 172) | CACGACGAGCCCATCAGC | CCTTCGTCCAGCCTCATCAG |
| GRMZM2G163418 | wrky38 (WRKY DNA-binding domain) | TCCTCCTCCTCGTCGACATA | CTCTAGCGCTCTTCCCTCCT |
| GRMZM2G152328 | actin | TTCCAGCCATCCTTCATCG | TCCTTGCTCATCCTGTCAG |

**Table S3** Gene annotations for 96 G×N interaction genes

| Gene id | Gene annotation (MaizeGDB) | Arabidopsis | | Class | TF |
| --- | --- | --- | --- | --- | --- |
| GRMZM2G003682 | Hs1pro-1, ABA induced-regulated-responsive-activated | | AtHSPRO2 | ABA |  |
| GRMZM2G046952 | GRAM domain-containing protein /ABA-responsive protein-related | | | ABA |  |
| GRMZM2G123394 | Hs1pro-1, ABA induced-regulated-responsive-activated | | AtHSPRO2 | ABA |  |
| GRMZM2G069146 | ereb115 (AP2-EREBP-transcription factor 115) | | CBF3, DREB1, DREB1A \| DREB1A | AP2-EREBP | √ |
| AC206951.3_FG017 | ereb182 (AP2-EREBP-transcription factor 182) | |  | AP2-EREBP | √ |
| GRMZM2G138396 | ereb211 (AP2-EREBP-transcription factor 211) | |  | AP2-EREBP | √ |
| GRMZM2G369472 | ereb172 (AP2-EREBP-transcription factor 172) | |  | AP2-EREBP | √ |
| GRMZM2G069126 | ereb23 (AP2-EREBP-transcription factor 23) | | CBF3, DREB1, DREB1A | AP2-EREBP | √ |
| GRMZM2G471304 | Auxin responsive protein | |  | ARF | √ |
| GRMZM2G392101 | crr2 (cytokinin response regulator2) | | ARR6 (Response Regulator 6) | ARR | √ |
| GRMZM2G078472 | Asparagine synthase | | AtASN1, DIN6 (Dark Inducible 6) | AS |  |
| GRMZM2G173124 | c3h47 (C3H-transcription factor 347), zinc finger | |  | C3H | √ |
| GRMZM2G155370 | CCT motif, C2C2(Zn) CO-like, Constans-like zinc finger family | | CIA2 (Chloroplast Import Apparatus 2) | CO-like | √ |
| AC234520.1_FG003 | glk9 (G2-like-transcription factor 9) | |  | G2-like | √ |
| GRMZM2G114184 | hagtf2 (GNAT-transcription factor 2) | | COP3, UNS2, HLS1 (Hookless 1) | GNAT | √ |
| GRMZM2G048297 | Homeobox-leucine zipper family protein | |  | Homeobox | √ |
| GRMZM2G098696 | hsftf7 (Heat shock factor protein 7) | | AtHSFB2B | HSF | √ |
| GRMZM2G177110 | lbd14 (LBD-transcription factor 14) | | LBD37 \| LBD38 | LBD | √ |
| GRMZM2G168665 | mterf1 ( mitochondrial transcription termination factor) | |  | mTERF | √ |
| GRMZM2G147346 | myb121 (MYB-transcription factor 121) | | AtMYB13 | MYB | √ |
| GRMZM2G406758 | U-box domain, protein degradation ubiquitin E3 RING | |  | ubiquitin E3 |  |
| GRMZM2G125034 | U-box domain, protein degradation ubiquitin E3 RING | |  | ubiquitin E3 |  |
| GRMZM2G027375 | U-box domain, protein degradation ubiquitin E3 RING | |  | ubiquitin E3 |  |
| GRMZM2G049185 | zinc finger family protein, protein degradation ubiquitin E3 RING | | | ubiquitin E3 |  |
| GRMZM2G148561 | wrky94 (WRKY DNA-binding domain) | AtWRKY15 | | WRKY | √ |
| GRMZM2G163418 | wrky38 (WRKY DNA-binding domain) |  | | WRKY | √ |
| GRMZM2G125653 | wrky53 (WRKY DNA-binding domain) | AtWRKY40 | | WRKY | √ |
| GRMZM2G161315 | zhd2 (ZF-HD-transcription factor 2) | AtHB34 (HOMEOBOX protein 34) | | ZF-HD | √ |
| GRMZM2G343157 | zim26 (ZIM-transcription factor 26) |  | | ZIM | √ |
| GRMZM2G001247 | Pollen proteins Ole e I like |  | |  |  |
| GRMZM2G141133 | propep4 (precursor elicitor peptide4) |  | |  |  |
| GRMZM2G180262 | VQ motif |  | |  |  |
| GRMZM2G030567 | no hits |  | |  |  |
| GRMZM2G069694 | Protein of unknown function (DUF506) |  | |  |  |
| GRMZM2G105184 | no hits |  | |  |  |
| GRMZM2G333448 | no hits |  | |  |  |
| AC203986.3_FG013 | no hits |  | |  |  |
| GRMZM2G323888 | no hits |  | |  |  |
| GRMZM2G129405 | no hits |  | |  |  |
| GRMZM2G111975 | Glycosyltransferase family 29 (sialyltransferase) |  | |  |  |
| GRMZM5G822237 | no hits |  | |  |  |
| GRMZM2G174396 | trpp8 (trehalose-6-phosphate phosphatase8) |  | |  |  |
| GRMZM2G056513 | no hits |  | |  |  |
| GRMZM2G343317 | no hits |  | |  |  |
| GRMZM2G134130 | Photosystem II protein Y (PsbY) | YCF32, PSBY (photosystem II BY) | |  |  |
| GRMZM2G070943 | Protein of unknown function (DUF740) |  | |  |  |
| GRMZM2G460566 | no hits |  | |  |  |
| GRMZM2G429955 | Chlorophyll A-B binding protein 48, chloroplast precursor | LHCB1.4, LHB1B1 | | | |
| GRMZM2G082487 | Protein phosphatase 2C |  | |  |  |
| GRMZM2G047422 | no hits |  | |  |  |
| GRMZM2G181227 | Enoyl-CoA hydratase/isomerase family | CHY1 | | |  |
| GRMZM2G337706 | Glutaredoxin |  | |  |  |
| GRMZM5G819464 | AAA-type ATPase family protein |  | |  |  |
| GRMZM2G377168 | no hits |  | |  |  |
| GRMZM2G450825 | Cytochrome C and Quinol oxidase polypeptide I | COX1 \| cytochrome c oxidase subunit 1 | |  |  |
| GRMZM2G011553 | harpin-induced protein |  | |  |  |
| GRMZM2G106819 | no hits |  | |  |  |
| GRMZM2G012728 | no hits |  | |  |  |
| GRMZM2G392863 | flower-specific gamma-thionin |  | |  |  |
| GRMZM2G103595 | DVL family |  | |  |  |
| GRMZM2G058884 | no hits |  | |  |  |
| GRMZM2G476637 | BTB/POZ domain, NPH3 family |  | |  |  |
| GRMZM2G052423 | cold acclimation protein cor413-pm1 | WCOR413, WCOR413-LIKE | |  |  |
| GRMZM2G458494 | Nodulin-like |  | |  |  |
| GRMZM2G340578 | Sodium/calcium exchanger protein | CAX7 (Calcium Exchanger 7) | |  |  |
| GRMZM2G131340 | no hits |  | |  |  |
| GRMZM2G066923 | Nucleoside transporter |  | |  |  |
| GRMZM2G031447 | Carboxylesterase family | AtCXE17 (Carboxyesterase 17) | |  |  |
| GRMZM2G354909 | short chain dehydrogenase |  | |  |  |
| GRMZM2G322950 | VQ motif |  | |  |  |
| GRMZM2G008839 | no hits |  | |  |  |
| GRMZM2G161274 | Ribonuclease T2 family |  | |  |  |
| GRMZM2G364748 | Glycosyl hydrolases family 16 |  | |  |  |
| GRMZM2G137535 | Glycosyl hydrolases family 17 | BG1 (BETA-1,3-Glucanase 1); hydrolase | |  |  |
| GRMZM2G020898 | Glycosyl hydrolases family 17 |  | |  |  |
| GRMZM2G346837 | VQ motif |  | |  |  |
| GRMZM2G325650 | Kelch motif, protein degradation ubiquitin E3 SCF F-box |  | |  |  |
| GRMZM2G097704 | triacylglycerol lipase like protein |  | |  |  |
| GRMZM5G855994 | U-box domain |  | |  |  |
| GRMZM2G125448 | Transferase family |  | |  |  |
| GRMZM2G369742 | VQ motif |  | |  |  |
| GRMZM2G093951 | Late embryogenesis abundant protein |  | |  |  |
| AC212023.4_FG004 | Plant invertase/pectin methylesterase inhibitor |  | |  |  |
| GRMZM2G324248 | no hits |  | |  |  |
| GRMZM2G155216 | Chlorophyll A-B binding protein | LHCB1.5, LHB1B2 | | | |
| GRMZM2G346861 | Thaumatin family |  | |  |  |
| GRMZM2G457929 | UDP-glucoronosyl and UDP-glucosyl transferase |  | |  |  |
| GRMZM2G046933 | no hits |  | |  |  |
| GRMZM2G046092 | no hits |  | |  |  |
| GRMZM2G067371 | Protein of unknown function (DUF1675) |  | |  |  |
| GRMZM2G308595 | NUDIX domain | AtNUDT21 (Nudix hydrolase homolog 21) | |  |  |
| GRMZM2G133819 | Primase zinc finger | MCM10 | |  |  |
| GRMZM2G371079 | no hits |  | |  |  |
| GRMZM2G120304 | stress responsive protein |  | |  |  |
| GRMZM2G124066 | Transferase family |  | |  |  |
| GRMZM2G090626 | no hits |  | |  |  |

**Table S4** GO terms with significant enrichments (FDR<0.05) for 96 G×N interaction genes

| GO term | Ontology | Description | Number in input list | Number in BG/Ref | p-value | FDR | Entries |
| --- | --- | --- | --- | --- | --- | --- | --- |
| GO:0080090 | P | regulation of primary metabolic process | 12 | 2312 | 0.0013 | 0.022 | GRMZM2G125653 GRMZM2G392101 GRMZM2G346861 AC206951.3_FG017 GRMZM2G069146 GRMZM2G148561 GRMZM2G069126 GRMZM2G098696 GRMZM2G369472 GRMZM2G163418 GRMZM2G138396 GRMZM2G131340 |
| GO:0019222 | P | regulation of metabolic process | 12 | 2373 | 0.0016 | 0.022 | GRMZM2G125653 GRMZM2G392101 GRMZM2G346861 AC206951.3_FG017 GRMZM2G069146 GRMZM2G148561 GRMZM2G069126 GRMZM2G098696 GRMZM2G369472 GRMZM2G163418 GRMZM2G138396 GRMZM2G131340 |
| GO:0031326 | P | regulation of cellular biosynthetic process | 12 | 2210 | 0.00086 | 0.022 | GRMZM2G125653 GRMZM2G392101 GRMZM2G346861 AC206951.3_FG017 GRMZM2G069146 GRMZM2G148561 GRMZM2G069126 GRMZM2G098696 GRMZM2G369472 GRMZM2G163418 GRMZM2G138396 GRMZM2G131340 |
| GO:0031323 | P | regulation of cellular metabolic process | 12 | 2250 | 0.001 | 0.022 | GRMZM2G125653 GRMZM2G392101 GRMZM2G346861 AC206951.3_FG017 GRMZM2G069146 GRMZM2G148561 GRMZM2G069126 GRMZM2G098696 GRMZM2G369472 GRMZM2G163418 GRMZM2G138396 GRMZM2G131340 |
| GO:0045449 | P | regulation of transcription | 12 | 2172 | 0.00074 | 0.022 | GRMZM2G125653 GRMZM2G392101 GRMZM2G346861 AC206951.3_FG017 GRMZM2G069146 GRMZM2G148561 GRMZM2G069126 GRMZM2G098696 GRMZM2G369472 GRMZM2G163418 GRMZM2G138396 GRMZM2G131340 |
| GO:0019219 | P | regulation of nucleobase, nucleoside, nucleotide and nucleic acid metabolic process | 12 | 2192 | 0.0008 | 0.022 | GRMZM2G125653 GRMZM2G392101 GRMZM2G346861 AC206951.3_FG017 GRMZM2G069146 GRMZM2G148561 GRMZM2G069126 GRMZM2G098696 GRMZM2G369472 GRMZM2G163418 GRMZM2G138396 GRMZM2G131340 |
| GO:0010468 | P | regulation of gene expression | 12 | 2216 | 0.00088 | 0.022 | GRMZM2G125653 GRMZM2G392101 GRMZM2G346861 AC206951.3_FG017 GRMZM2G069146 GRMZM2G148561 GRMZM2G069126 GRMZM2G098696 GRMZM2G369472 GRMZM2G163418 GRMZM2G138396 GRMZM2G131340 |
| GO:0060255 | P | regulation of macromolecule metabolic process | 12 | 2335 | 0.0014 | 0.022 | GRMZM2G125653 GRMZM2G392101 GRMZM2G346861 AC206951.3_FG017 GRMZM2G069146 GRMZM2G148561 GRMZM2G069126 GRMZM2G098696 GRMZM2G369472 GRMZM2G163418 GRMZM2G138396 GRMZM2G131340 |
| GO:0009889 | P | regulation of biosynthetic process | 12 | 2210 | 0.00086 | 0.022 | GRMZM2G125653 GRMZM2G392101 GRMZM2G346861 AC206951.3_FG017 GRMZM2G069146 GRMZM2G148561 GRMZM2G069126 GRMZM2G098696 GRMZM2G369472 GRMZM2G163418 GRMZM2G138396 GRMZM2G131340 |
| GO:0051171 | P | regulation of nitrogen compound metabolic process | 12 | 2208 | 0.00085 | 0.022 | GRMZM2G125653 GRMZM2G392101 GRMZM2G346861 AC206951.3_FG017 GRMZM2G069146 GRMZM2G148561 GRMZM2G069126 GRMZM2G098696 GRMZM2G369472 GRMZM2G163418 GRMZM2G138396 GRMZM2G131340 |
| GO:0051252 | P | regulation of RNA metabolic process | 9 | 1434 | 0.0016 | 0.022 | GRMZM2G069126 GRMZM2G392101 GRMZM2G346861 AC206951.3_FG017 GRMZM2G069146 GRMZM2G098696 GRMZM2G369472 GRMZM2G138396 GRMZM2G131340 |
| GO:0006355 | P | regulation of transcription, DNA-dependent | 9 | 1432 | 0.0016 | 0.022 | GRMZM2G069126 GRMZM2G392101 GRMZM2G346861 AC206951.3_FG017 GRMZM2G069146 GRMZM2G098696 GRMZM2G369472 GRMZM2G138396 GRMZM2G131340 |
| GO:0010556 | P | regulation of macromolecule biosynthetic process | 12 | 2210 | 0.00086 | 0.022 | GRMZM2G125653 GRMZM2G392101 GRMZM2G346861 AC206951.3_FG017 GRMZM2G069146 GRMZM2G148561 GRMZM2G069126 GRMZM2G098696 GRMZM2G369472 GRMZM2G163418 GRMZM2G138396 GRMZM2G131340 |
| GO:0006350 | P | transcription | 12 | 2389 | 0.0017 | 0.022 | GRMZM2G125653 GRMZM2G392101 GRMZM2G346861 AC206951.3_FG017 GRMZM2G069146 GRMZM2G148561 GRMZM2G069126 GRMZM2G098696 GRMZM2G369472 GRMZM2G163418 GRMZM2G138396 GRMZM2G131340 |
| GO:0032774 | P | RNA biosynthetic process | 9 | 1508 | 0.0023 | 0.026 | GRMZM2G069126 GRMZM2G392101 GRMZM2G346861 AC206951.3_FG017 GRMZM2G069146 GRMZM2G098696 GRMZM2G369472 GRMZM2G138396 GRMZM2G131340 |
| GO:0006351 | P | transcription, DNA-dependent | 9 | 1506 | 0.0022 | 0.026 | GRMZM2G069126 GRMZM2G392101 GRMZM2G346861 AC206951.3_FG017 GRMZM2G069146 GRMZM2G098696 GRMZM2G369472 GRMZM2G138396 GRMZM2G131340 |
| GO:0065007 | P | biological regulation | 18 | 4740 | 0.0024 | 0.026 | GRMZM2G333448 GRMZM2G476637 GRMZM2G125653 GRMZM2G141133 GRMZM2G392101 GRMZM2G346861 AC206951.3_FG017 GRMZM2G069146 GRMZM2G148561 GRMZM2G134130 GRMZM2G337706 GRMZM2G069126 GRMZM2G098696 GRMZM2G369472 GRMZM2G377168 GRMZM2G163418 GRMZM2G138396 GRMZM2G131340 |
| GO:0016070 | P | RNA metabolic process | 10 | 1932 | 0.0035 | 0.036 | GRMZM2G069126 GRMZM2G392101 GRMZM2G346861 AC206951.3_FG017 GRMZM2G069146 GRMZM2G098696 GRMZM2G369472 GRMZM2G161315 GRMZM2G138396 GRMZM2G131340 |
| GO:0050794 | P | regulation of cellular process | 14 | 3392 | 0.0041 | 0.039 | GRMZM2G333448 GRMZM2G125653 GRMZM2G392101 GRMZM2G346861 AC206951.3_FG017 GRMZM2G069146 GRMZM2G148561 GRMZM2G337706 GRMZM2G069126 GRMZM2G098696 GRMZM2G369472 GRMZM2G163418 GRMZM2G138396 GRMZM2G131340 |
| GO:0003700 | F | transcription factor activity | 9 | 923 | 6.50E-05 | 0.0022 | GRMZM2G069126 GRMZM2G125653 AC206951.3_FG017 GRMZM2G069146 GRMZM2G148561 GRMZM2G098696 GRMZM2G369472 GRMZM2G163418 GRMZM2G138396 |
| GO:0030528 | F | transcription regulator activity | 10 | 1432 | 0.00037 | 0.0061 | GRMZM2G125653 GRMZM2G392101 GRMZM2G069126 AC206951.3_FG017 GRMZM2G069146 GRMZM2G148561 GRMZM2G098696 GRMZM2G369472 GRMZM2G163418 GRMZM2G138396 |
| GO:0016874 | F | ligase activity | 5 | 453 | 0.0019 | 0.021 | GRMZM2G125034 GRMZM2G027375 GRMZM2G161315 GRMZM2G406758 GRMZM2G078472 |

**Table S5** Pathway annotation for 96 G×N interaction genes

| **Gene id** | **Bincode** | **MapMan pathway description** |
| --- | --- | --- |
| GRMZM2G429955 | 1.1.1.1 | PS.lightreaction.photosystem II.LHC-II |
| GRMZM2G155216 | 1.1.1.1 | PS.lightreaction.photosystem II.LHC-II |
| GRMZM2G134130 | 1.1.1.2 | PS.lightreaction.photosystem II.PSII polypeptide subunits |
| GRMZM2G046092 | 1.1.1.2 | PS.lightreaction.photosystem II.PSII polypeptide subunits |
| GRMZM2G174396 | 3.2.2 | minor CHO metabolism.trehalose.TPP |
| GRMZM2G450825 | 9.7 | mitochondrial electron transport / ATP synthesis.cytochrome c oxidase |
| GRMZM2G364748 | 10.7 | cell wall.modification |
| GRMZM2G097704 | 11.9.2.1 | lipid metabolism.lipid degradation.lipases.triacylglycerol lipase |
| GRMZM2G031447 | 11.9.3.2 | lipid metabolism.lipid degradation.lysophospholipases.carboxylesterase |
| GRMZM2G078472 | 13.1.3.1 | amino acid metabolism.synthesis.aspartate family.asparagine |
| GRMZM2G181227 | 13.2.6.3 | amino acid metabolism.degradation.aromatic aa.tryptophan |
| GRMZM2G125448 | 16.2 | secondary metabolism.phenylpropanoids |
| GRMZM2G124066 | 16.2 | secondary metabolism.phenylpropanoids |
| GRMZM2G046952 | 17.1.3 | hormone metabolism.abscisic acid.induced-regulated-responsive-activated |
| GRMZM2G471304 | 17.2.3 | hormone metabolism.auxin.induced-regulated-responsive-activated |
| GRMZM2G093951 | 20.1 | stress.biotic |
| GRMZM2G346861 | 20.1 | stress.biotic |
| GRMZM2G098696 | 20.2.1 | stress.abiotic.heat |
| GRMZM2G052423 | 20.2.2 | stress.abiotic.cold |
| GRMZM2G337706 | 21.4 | redox.glutaredoxins |
| AC212023.4_FG004 | 26.18 | misc.invertase/pectin methylesterase inhibitor family protein |
| GRMZM2G457929 | 26.2 | misc.UDP glucosyl and glucoronyl transferases |
| GRMZM2G354909 | 26.22 | misc.short chain dehydrogenase/reductase (SDR) |
| GRMZM2G114184 | 26.24 | misc.GCN5-related N-acetyltransferase |
| GRMZM2G137535 | 26.4 | misc.beta 1,3 glucan hydrolases |
| GRMZM2G020898 | 26.4 | misc.beta 1,3 glucan hydrolases |
| GRMZM2G161274 | 27.1.19 | RNA.processing.ribonucleases |
| GRMZM2G173124 | 27.3.12 | RNA.regulation of transcription.C3H zinc finger family |
| AC234520.1_FG003 | 27.3.20 | RNA.regulation of transcription.G2-like transcription factor family, GARP |
| GRMZM2G048297 | 27.3.22 | RNA.regulation of transcription.HB,Homeobox transcription factor family |
| GRMZM2G147346 | 27.3.25 | RNA.regulation of transcription.MYB domain transcription factor family |
| GRMZM2G069146 | 27.3.3 | RNA.regulation of transcription.AP2/EREBP, APETALA2/Ethylene-responsive element binding protein family |
| AC206951.3_FG017 | 27.3.3 | RNA.regulation of transcription.AP2/EREBP, APETALA2/Ethylene-responsive element binding protein family |
| GRMZM2G138396 | 27.3.3 | RNA.regulation of transcription.AP2/EREBP, APETALA2/Ethylene-responsive element binding protein family |
| GRMZM2G369472 | 27.3.3 | RNA.regulation of transcription.AP2/EREBP, APETALA2/Ethylene-responsive element binding protein family |
| GRMZM2G069126 | 27.3.3 | RNA.regulation of transcription.AP2/EREBP, APETALA2/Ethylene-responsive element binding protein family |
| GRMZM2G148561 | 27.3.32 | RNA.regulation of transcription.WRKY domain transcription factor family |
| GRMZM2G163418 | 27.3.32 | RNA.regulation of transcription.WRKY domain transcription factor family |
| GRMZM2G125653 | 27.3.32 | RNA.regulation of transcription.WRKY domain transcription factor family |
| GRMZM2G177110 | 27.3.37 | RNA.regulation of transcription.AS2,Lateral Organ Boundaries Gene Family |
| GRMZM2G392101 | 27.3.5 | RNA.regulation of transcription.ARR |
| GRMZM2G155370 | 27.3.7 | RNA.regulation of transcription.C2C2(Zn) CO-like, Constans-like zinc finger family |
| GRMZM2G161315 | 27.3.80 | RNA.regulation of transcription.zf-HD |
| GRMZM2G168665 | 27.3.99 | RNA.regulation of transcription.unclassified |
| GRMZM2G067371 | 29.3.1 | protein.targeting.nucleus |
| GRMZM2G082487 | 29.4 | protein.postranslational modification |
| GRMZM2G406758 | 29.5.11.4.2 | protein.degradation.ubiquitin.E3.RING |
| GRMZM2G125034 | 29.5.11.4.2 | protein.degradation.ubiquitin.E3.RING |
| GRMZM2G027375 | 29.5.11.4.2 | protein.degradation.ubiquitin.E3.RING |
| GRMZM5G855994 | 29.5.11.4.2 | protein.degradation.ubiquitin.E3.RING |
| GRMZM2G325650 | 29.5.11.4.3.2 | protein.degradation.ubiquitin.E3.SCF.FBOX |
| GRMZM5G819464 | 29.5.9 | protein.degradation.AAA type |
| GRMZM2G111975 | 29.7 | protein.glycosylation |
| GRMZM2G476637 | 30.11 | signalling.light |
| GRMZM2G458494 | 33.99 | development.unspecified |
| GRMZM2G066923 | 34.14 | transport.unspecified cations |
| GRMZM2G340578 | 34.21 | transport.calcium |
| GRMZM2G001247 | 35.2 | not assigned.unknown |
| GRMZM2G141133 | 35.2 | not assigned.unknown |
| GRMZM2G180262 | 35.2 | not assigned.unknown |
| GRMZM2G030567 | 35.2 | not assigned.unknown |
| GRMZM2G069694 | 35.2 | not assigned.unknown |
| GRMZM2G105184 | 35.2 | not assigned.unknown |
| GRMZM2G333448 | 35.2 | not assigned.unknown |
| GRMZM2G003682 | 35.2 | not assigned.unknown |
| AC203986.3_FG013 | 35.2 | not assigned.unknown |
| GRMZM2G323888 | 35.2 | not assigned.unknown |
| GRMZM2G129405 | 35.2 | not assigned.unknown |
| GRMZM5G822237 | 35.2 | not assigned.unknown |
| GRMZM2G056513 | 35.2 | not assigned.unknown |
| GRMZM2G343317 | 35.2 | not assigned.unknown |
| GRMZM2G070943 | 35.2 | not assigned.unknown |
| GRMZM2G460566 | 35.2 | not assigned.unknown |
| GRMZM2G047422 | 35.2 | not assigned.unknown |
| GRMZM2G377168 | 35.2 | not assigned.unknown |
| GRMZM2G011553 | 35.2 | not assigned.unknown |
| GRMZM2G106819 | 35.2 | not assigned.unknown |
| GRMZM2G012728 | 35.2 | not assigned.unknown |
| GRMZM2G392863 | 35.2 | not assigned.unknown |
| GRMZM2G103595 | 35.2 | not assigned.unknown |
| GRMZM2G058884 | 35.2 | not assigned.unknown |
| GRMZM2G123394 | 35.2 | not assigned.unknown |
| GRMZM2G131340 | 35.2 | not assigned.unknown |
| GRMZM2G343157 | 35.2 | not assigned.unknown |
| GRMZM2G322950 | 35.2 | not assigned.unknown |
| GRMZM2G008839 | 35.2 | not assigned.unknown |
| GRMZM2G346837 | 35.2 | not assigned.unknown |
| GRMZM2G049185 | 35.2 | not assigned.unknown |
| GRMZM2G369742 | 35.2 | not assigned.unknown |
| GRMZM2G324248 | 35.2 | not assigned.unknown |
| GRMZM2G046933 | 35.2 | not assigned.unknown |
| GRMZM2G308595 | 35.2 | not assigned.unknown |
| GRMZM2G133819 | 35.2 | not assigned.unknown |
| GRMZM2G371079 | 35.2 | not assigned.unknown |
| GRMZM2G090626 | 35.2 | not assigned.unknown |
| GRMZM2G120304 | 35.2 | not assigned.unknown |
